# Editorial: Neuropsychological mechanisms underlying risk, resilience, and intervention response in youth substance use

**DOI:** 10.3389/fpsyg.2026.1901240

**Published:** 2026-06-30

**Authors:** Iliyan Ivanov, James Blair, Christopher J. Hammond

**Affiliations:** 1 Department of Psychiatry, Icahn School of Medicine at Mount Sinai, New York, NY, United States; 2 Department of Psychiatry, Virginia Commonwealth University School of Medicine, Richmond, VA, United States; 3 Department of Psychiatry and Behavioral Sciences, Johns Hopkins University School of Medicine, Baltimore, MD, United States

**Keywords:** editorial/personal viewpoint, environmental factors, resilience, risk factors, substance use

It is our great pleasure to share this Research Topic of *Frontiers in Psychiatry* on Mechanisms underlying risk, resilience and intervention response in youth substance use. Our goal was to present a variety of papers from diverse research groups that will cover a broad spectrum of topics reflecting the ever-expanding field of research on risk and resilience with respect to substance use and addictive disorders (SU/ADs) in adolescence. The resulting Research Topic includes diverse papers spanning commentary, methods papers, comprehensive reviews, and reports on novel investigations from both experimental studies and big data analyses from author groups from around the world. The substance-related topics of these papers cover both substance and behavioral addictions. Collectively, the Research Topic addresses a number of aspects critical to this important public health topic. Moreover, we wish to present these reports in a developmentally-sensitive fashion adhering to the concept that SU/ADs are not episodic conditions but have diverse and lasting effects from “cradle to grave.” Lastly, this Research Topic is a testimony to the progress that professionals in the field of child and adolescent psychiatry and allied professions have made in studying the neurobiological, psychological, and environmental factors underlying risk vs. resilience toward adolescent SU/ADs and applying evidence-based treatments for these conditions.

Several of the studies considered risk factors for addiction. This included psychosocial variables (Aloi et al.; Bador et al.; Zhan and Zhu). Two of these papers highlighted the pernicious impact of adverse childhood experience with respect to future risk for substance use (Aloi et al.; Bador et al.). Interestingly, Aloi et al. took a mechanistic approach with their results showing that exposure to violence and extreme threats were associated with alterations in neural responses to anticipated reward during risky vs. safe decision-making in prefrontal and reward regions that prospectively predicted problematic substance use at follow-up. In their reviews of the literature, both Bador et al. and Zhan and Zhu examined psychosocial risk factors more generally as well as personality variables and revealed some degree of commonality of risk factors for both risk for substance use (Bador et al.) and short form video addiction (Zhan and Zhu). Balhara et al. highlighted the emerging role of artificial intelligence (AI) and machine learning in the field, outlining a protocol for future work applying AI prediction models to predict problematic use of digital technologies (termed “digital addiction”).

Two other papers concentrated on brain-based risk factors for addictive disorders. These included a commentary article by Hussin describing the unique risk-inducing context of the prison system and presenting a neuropsychological framework for addiction vulnerability among people who are incarcerated and an original research article by Adams et al. testing the relative influence of response inhibition as indexed by neurobehavioral responses on the Stop Signal task relate to alcohol experimentation during early adolescence. Interestingly, in the Adams et al. study, self-report measures of impulsivity and behavioral performance on the stop signal task did not differentiate adolescents who experimented with alcohol from those who did not, however, neural response patterns during inhibition from the stop signal task did.

This grouping of articles is also notable for its global viewpoint and broad focus covering both substance use and related disorders and behavioral addictions and including author groups from around the world. Four of the included publications had a multinational focus, examining global trends in substance use patterns or comparing risk factors or health correlates of addictive disorders across countries or geographic regions (Venero-Hidalgo et al.; Bador et al.; Su et al.; Zhan and Zhu). Additionally, three of the included publications examined health effects of substance use and behavioral addictions. In a systematic review Venero-Hidalgo et al., investigated neuropsychological effects of cannabis use with particular interest on the influence of age across various geographical locations including data form Americas, Europe, and Asia. Their results suggest that younger age was more consistently associated with the effects of cannabis on cognition while geographical location played a role in relation to study types and methodologies. Another reported by Su et al., used data form the Global Burden of Diseases, Injuries, and Risk Factors Study (GBD) 2021 to find continuing escalation of the rates of cannabis and opioid use disorders among young adults. These trends are linked to increased rates of liver cancer mortality and disability-adjusted life years. Finally, one study by Wang et al., reported on the contribution of excessive use of smart phones to depressive symptoms in medical students possibly by worsening of self-acceptance and contributing to higher levels of loneliness.

In summary, we are presenting a Research Topic incorporating the work of researchers from wide background in respect to both expertise and location. We believe the selected articles bring new information and insight on the topic of “*Neurobiological, Psychological, and Environmental Factors Underlying Risk Versus Resilience for Substance Use and Addictive Disorders in Youth*.”

